# Reducing suicidal ideation among Turkish migrants in the Netherlands and in the UK: the feasibility of a randomised controlled trial of a guided online intervention

**DOI:** 10.1186/s40814-021-00772-9

**Published:** 2021-01-25

**Authors:** Ozlem Eylem, Annemieke van Straten, Leonore de Wit, Shanaya Rathod, Kamaldeep Bhui, Ad J. F. M. Kerkhof

**Affiliations:** 1 Department of Clinical Psychology, Vrije Universiteit Amsterdam, 7 van der Boechorststraat, Amsterdam, 1081 BT Netherlands; 2 Amsterdam Institute of Public Health, Amsterdam, Netherlands; 3 Centre for Psychiatry, Wolfson Institute of Preventive Medicine, Charterhouse Square, London, EC1M 6BQ UK; 4 Southern Health NHS Foundation Trust, Southampton, UK; 5 Nuffield Department of Primary Care Health Sciences, University of Oxford, Oxford, OX2 6GG UK

**Keywords:** e-mental health, Cultural adaptation, Suicidal ideation, Turkish migrants, Feasibility, RCT

## Abstract

**Background:**

The evidence for the effectiveness of e-mental health interventions among ethnic minorities is still preliminary. This mixed methods study investigates the feasibility of a culturally adapted, guided online intervention with the intention to understand how it works and for whom to inform refinement. It also examines its likely effectiveness in reducing suicidal ideation when compared with the treatment as usual.

**Methods:**

Turkish migrants with mild to moderate suicidal thoughts were recruited from the general population using social media and newspaper advertisements. The intervention group obtained direct access to a 6-week guided online intervention while participants in the waiting list condition had to wait for 6 weeks. The intervention is based on an existing online intervention and was culturally adapted. Participants in both conditions completed baseline, post-test, and follow-up questionnaires on suicidal ideation (primary outcome), depression, worrying, hopelessness, suicide attempt and self-harm, acculturation, quality of life, and usability. In addition, participants were interviewed to examine the feasibility and mechanisms of action in more depth. The responses were analysed by inductive thematic analysis.

**Results:**

Eighty-five people signed up via the study website, and we included 18 (10 intervention, 8 waitlist control). While the therapeutic benefits were emphasised (e.g. feeling connected with the intervention), the feasibility was judged to be low. The main reasons given were not having severe suicidal thoughts and not being represented by the culturally adapted intervention. No suicide attempts were recorded during the study. The suicidal ideation, depression, and hopelessness scores were improved in both groups.

**Conclusion:**

Although intended to be a definitive trial, the current study became a feasibility study with process evaluation to understand the components and how they operate. The online intervention was not superior to the control condition. Future studies need to attend the implementation issues raised including measures of stigma, acculturation, and careful cultural adaptations alongside more attention to coaching and relational support. They should also consider how to improve engagement alongside selection of those who are motivated to use online interventions and offer alternatives for those who are not.

**Trial registration:**

Netherlands Trial Register, NTR5028. Registered on 1 March 2015

**Supplementary Information:**

The online version contains supplementary material available at 10.1186/s40814-021-00772-9.

## Key messages regarding feasibility

- There is growing evidence for the effectiveness of e-mental health in reducing suicidal thoughts. However, it is not known whether e-mental health could improve engagement with ethnic minorities in their help-seeking process for suicidal thoughts.
- This feasibility study identified implementation issues compromising the usability of culturally adapted e-mental health in daily life. Those with mild suicidal ideation and those who were not relating to the culturally adapted content (e.g. specific cultural case examples) emphasised not being represented by the content of the intervention.
- This study and the previous trials seem to suggest that the intervention needs further work and refinement and there should be more feasibility and exploratory trials of a modified intervention, refine it iteratively with feedback. More attention should be paid on how to improve engagement alongside selection of those who are motivated to use online interventions and offer alternatives for those who are not.

## Background

Suicide is a global public health problem with enormous consequences at individual and societal levels [1]. The international lifetime prevalence of suicidal ideation, plans and attempts in general population is 9.2%, 3.1% and 2.7%, respectively [2]. Members of some ethnic groups are at higher risk for suicidal behaviours compared to others [3]. In Europe, Turkish populations are among the largest ethnic minority populations and they have disproportionate rates of suicidal behaviours compared to the ethnic majorities in their respective host countries [4]. In the Netherlands, there is an elevated risk of suicidal ideation in Turkish adolescents (38.1%) when compared with ethnic Dutch (17.9%) adolescents [5]. Suicide attempt risk is increased 2–5-fold among Turkish migrant women aged 14 and 25 when compared with the same aged and locally born women in the Netherlands [6], Germany [7] and Switzerland [8]. It is difficult to establish the accurate statistics in the UK due to the absence of a separate category for Turkish people in the national statistics [9, 10]. The causes of the increased prevalence rates are not clear yet. Some have argued that this might be associated with gender-related factors such as domestic violence and honour-related violence (e.g. being forced into an unwanted marriage), which are important life events among women who are presenting suicidal behaviours worldwide [11, 12]. Others have stressed that people might encounter difficulties in their adaptation process to host countries, and interpersonal and structural discrimination within institutions, contributing to the elevated risk of suicidal behaviour [12, 13].

Existing guidelines for treating suicidal behaviour in the UK and in the Netherlands recommend Cognitive Behavioural Therapy (CBT) and CBT-based interventions, such as Dialectical Behavioural Therapy (DBT), in managing suicidal behaviour which are delivered face-to-face [14, 15]. These interventions are based on a general cognitive model which is expanded to suicidal behaviours [15–18]. How one interprets a situation determines emotional and behavioural reactions to those situations [17, 18]. Interpretation is distorted by errors in thinking such as overgeneralisation and emotional reasoning. CBT interventions aim to restructure thinking errors with the intention to help individuals to make sense of situations from a more realistic point of view [17]. The efficacy of the Mentalization-Based Treatment (MBT) has also been demonstrated in several randomised controlled trials (RCTs) [19, 20] and longitudinal studies [21] among adult and adolescent populations. Mentalisation is the capacity to understand actions in terms of thoughts and feelings [19]. When mentalising is compromised in interpersonal relationships, negative thoughts are experienced in greater intensity leading to an urgent need for distraction. In this context, suicidal behaviours may serve as distraction [19].

In recent years, online interventions have been introduced into the mental health services as an addition to, or alternative for, the preceding interventions. These interventions can be delivered through personal computers (PC), mobile phones or tablets and can be either guided or unguided. Guidance can be delivered by a clinician or a trained coach [22] and is aimed at motivating patients, to explain things which are unclear and to provide feedback on the content of the sessions [22]. Unguided e-mental health interventions may involve automated feedback but do not provide any professional support related to the therapeutic content [22]. Guided interventions are often more effective than unguided interventions [23], but unguided interventions are more scalable and can reach larger groups of people [24].

There are certain disadvantages of the former face-to-face interventions which are especially true for ethnic minorities [3, 22]. Cultural barriers such as stigma and shame attached to suicide might prevent them from utilising these interventions [22, 23]. Additionally, poor language proficiency of the help-seeker and the cultural mismatch between the mental health professional and the help-seeker often result in communication barriers during the help-seeking process [23–25]. It is therefore a global challenge to optimise these interventions for migrants and ethnic minorities [26].

There is growing evidence supporting the value of online interventions in engaging with people from across the life span and from different ethnic backgrounds in treatment of common mental disorders such as depression and anxiety [27–29]. There are also several randomised controlled trials (RCTs) testing the effectiveness of online interventions for people with suicidal ideation [30–32]. Recently, meta-analytic studies indicated significant beneficial treatment effect of those interventions compared to treatment as usual (*g* = − 0.26; 95% CI − 0.48, − 0.03) [33]. These studies however have been carried out in general populations. Their effectiveness for even more vulnerable populations, for instance, migrants and ethnic minorities with suicidal ideation, is lacking [34–37].

In this study, we have used an e-mental health intervention for suicidal ideation which was developed for the general population in the Netherlands by van Spijker and colleagues [38]. We have adapted this intervention for the Turkish migrants in the Netherlands and in the UK [23, 39]. Cultural adaptation is defined as ‘the systematic modification of an evidence-based treatment or intervention protocol to consider language, culture, and context in such a way that it is compatible with the individual’s cultural patterns, meanings, and values’ [40]. CBT interventions offer flexibility to be adapted according to the needs and expectations of a diverse help-seeker population in multicultural health care [26]. Culturally adapted online interventions seem promising for ethnic minorities in general [41] and for Turkish migrants specifically [42, 43].

The objectives of this study are twofold: (1) to investigate the feasibility of the adapted online intervention among Turkish migrants in the UK and in the Netherlands; (2) and to investigate the likely effects of the culturally adapted online intervention in reducing suicidal ideation when compared with the treatment as usual.

## Methods

### Design, settings and participants

This study is a RCT in which patients were randomised to the guided online intervention or to a waitlist control group. The control group had direct access to a website with psycho-education about the reasons for suicidal thoughts, risk factors and where to seek help (e.g. 113 online, Samaritans, mental health organisations) and could start with the intervention after the post-test measurement at 6 weeks. As part of the feasibility, semi-structured telephone interviews were held at 6 weeks with the intention to obtain in-depth information about how the intervention was implemented and the contextual factors related with the uptake of the intervention in daily life. Participants were recruited between January 2017 and October 2018.

Recruitment took place among the Turkish-speaking population (i.e. people of Turkish, Kurdish and Turkish Cypriot background) [23, 44] in the Netherlands and in the UK. Participants were recruited from the general population through newspaper advertisements, TV programmes, banners on relevant websites (e.g. of community and health organisations), social media and public events. These events were organised in collaboration with the community organisations representing the population of interest.

Eligible participants were 18 years and older, had Turkish background (being born in Turkey or having at least one parent being born in Turkey), had a suicidal ideation score of 1 or higher on the Beck Scale for Suicide Ideation (BSS), had access to a PC and Internet and consented to provide their name, telephone number, e-mail address and the e-mail of their general practitioner (GP). These data were needed for our safety procedure. Exclusion criteria were (1) being younger than 18 at the time of the study, (2) not having a Turkish background, (3) not being registered with a GP, (4) not residing in the Netherlands and/or in the UK where the recruitment took place, (5) being computer illiterate and (6) not having access to the Internet. Already receiving help, regardless of the source, was not an exclusion criterion.

A study website (http://kiymacanina.org/) was created where potential participants could find more information about the study and register in Turkish, English and Dutch. Those who registered received an e-mail with further information, an informed consent form and a link to the baseline questionnaire. Those who returned the informed consent, provided their contact details and those of their GPs, filled out the baseline questionnaire and did not fulfil our exclusion criteria were included. The randomisation scheme was derived using random allocation software by an independent researcher. Randomisation was stratified for the UK and the Netherlands and took place in a 1:1 ratio. The outcome was communicated to the participant by e-mail with either a log-in code for the intervention or a link to a website with general information on suicide for the waitlist control group. The study protocol is described in more detail elsewhere [39] and was registered in the Netherlands Trial Register NTR5028 (see https://www.trialregister.nl/trial/4926).

### Ethics statement

This study was approved by the Medical Ethics Committee of the VU University Medical Centre in the Netherlands (registration number 2014. 187) and by the Queen Mary University of London Research Ethics Committee in the UK (registration number QMERC2014/46). Written informed consent of participants was obtained after the study, and all procedures had been fully explained in writing. Participants could ask questions by e-mail or telephone if wanted.

### Safety

As this study involved vulnerable people who are at risk of suicide, a safety protocol was used [39]. In summary, every participant (in both conditions) was asked to fill out the Beck Suicidal Ideation Scale (BSS) once in 2 weeks throughout their participation. If a participant exceeded the cut-off score of 29 on the BSS, we were obligated to phone the participant to perform a risk assessment. If deemed necessary, the next step of action was contacting their GP. The phone calls were going to be made by a psychologist in the research team under the supervision of a licenced clinical psychologist in the Netherlands and a licenced consultant psychiatrist in the UK who were both experienced in suicide prevention. As a last resort, participants’ GPs were also going to be contacted if they could not be reached.

### Intervention

The original version of the intervention is developed by van Spijker and colleagues [45]. RCTs investigating the unguided version of this intervention showed its effectiveness in reducing suicidal ideation compared to the treatment as usual in general Dutch (*d* = 0.2) [30] and Belgian populations (*d* = 0.34) [32], but it showed no effect when implemented in a general Australian population [31].

The intervention is based on the CBT framework [46]. Within this framework, some mindfulness exercises were also included. The main principle is that worry, rumination and repetitive suicidal ideation each produce obsessive attention to particular thoughts, sometimes resulting in a desire to end consciousness as a way to end the tantalising repetition of suicidal thoughts [45]. Thus, the aim of this intervention is to enhance controlled thinking (i.e. focusing on postponing worrisome thoughts to specific time-slots ‘worry times’ of the day and not thinking of these thoughts for the rest of the day).

The intervention consists of six modules: (1) the repetitive character of suicidal thoughts, (2) regulating intense emotions, (3) identifying automatic thoughts, (4) thinking patterns, (5) thought challenging, and (6) relapse prevention [38, 45]. Each module contains a theory section, a weekly assignment and several exercises. Weekly assignment is an essential element of the intervention encouraging participants to practice the techniques they learn from the intervention in daily life. For example, the first module explains that suicidal thoughts can develop out of self-protection, as keeping on living may seem worse than dying. Similarities between worry and suicidal thinking are also outlined. A weekly assignment involves tallying suicide-related thoughts to obtain an idea of how often these thoughts occur, while the exercises are aimed at learning to manage these repetitions better by introducing worry postponement. Participants are advised to do one module per week.

Guidance was available from coaches (2 in the UK and 2 in the Netherlands), who were supervised by a team of three experts including a psychologist, a licenced clinical psychologist (NL) and a consultant psychiatrist (UK). The coaches were bi-lingual (English-Turkish or Dutch-Turkish) and were either students at a Masters level or practitioners seeing patients. They received training about how the online intervention works, safety protocol, referral system in both countries and their roles and responsibilities while providing guidance to the participants. Regular supervision meetings through skype and/or e-mail communications were arranged.

Coaches provided online feedback to participants after their completion of each module. The aim of this personalised feedback was to help participants to understand the exercises and homework assignments as explained in the lessons. Moreover, it was used to motivate the participants to continue with the intervention.

### Cultural adaptation

We adapted this intervention linguistically and culturally for the Turkish migrant populations in line with the evidence indicating that interventions work better when adapted to local settings [41]. The decisions made during the cultural adaptation process are outlined in Additional file 1: appendix B. In summary, we used the ecological validity model of Bernal and colleagues [40]. This framework retains the core principles of CBT in order to preserve treatment validity but permits flexibility [26, 47]. The model delineates eight dimensions when culturally adapting an intervention: the use of appropriate language, persons (cultural similarities/differences between the client and clinician which shape the therapeutic relationship), metaphors (symbols and concepts), content (cultural knowledge), concepts (treatment concepts that are culturally congruent), goals (that support adaptive cultural values), methods (cultural enhancement of treatment methods) and context (consideration of acculturation, social context) [40] (see Additional file 1: Appendix B).

First, the intervention was forward translated and back-translated. The consistency of the adaptations in three languages (English, Dutch and Turkish) was checked by bi-lingual speakers. The cultural and linguistic adaptation was based on the results of 6 focus groups and 7 individual interviews with 38 Turkish-speaking lay people and 4 professionals living in the Netherlands and in the UK during the year 2014/2015 [23]. The key suggestions for the cultural adaptation of the intervention were enriching the content of the intervention with relevant case studies, including quotations from participants, who have found the programme useful, and reducing textual information and including more visuals such as pictures of nature and people who look happy, calm and/or relaxed [23]. In light of these, we made modifications in the concepts (see Additional file 1: Appendix B). For instance, we included some well-known idioms and metaphors describing psychological distress and suicide in Turkish language. Furthermore, modifications in the context included cultural case examples (see Additional file 1: Appendix B). Additionally, some theoretical modifications have also been made. In line with the well-documented evidence supporting the value of MBT [19–21] for instance, a well-known MBT based exercise called ‘safe place’ was incorporated into the crisis plan in the intervention. This exercise uses guided imaginary and encourages people to create an imaginary safe place that they could visit whenever they feel the need to be grounded [19] (see Additional file 1: appendix B).

### Deviations from the study protocol

The sample size of this RCT was calculated based on the expected effect of the primary outcome measure: the reduction in frequency and intensity of suicidal thoughts as measured with the BSS (*d* = 0.40). The rationale for this decision was based on the trial of van Spijker who found an effect size of *d* = 0.2 for an unguided version of the treatment [30]. We expected a higher effect size since we provided personal coaching which generally leads to higher effect sizes [28]. Based on a power of 0.80 and an alpha of 0.05, 100 participants were needed in each condition. Given the expected drop-out of 30%, the total sample size was determined as being 286 [39].

Although intended to be a definitive trial, at best, it became a feasibility study with process evaluation to understand the components and how they operate. We conducted in-depth interviews with the participants who completed the intervention and consented to be interviewed on feasibility issues. Furthermore, we have excluded the following questionnaires from the study: Suicidal Ideation Attribution Scale, the Hospital Anxiety and Depression Scale and an item measuring the satisfaction with the treatment. This was done to reduce the burden on participants and to increase their motivation to fill out our questionnaires. Moreover, we did not include 3-month follow-up assessments (T4) in the analyses and have done completer analyses. The reason was the underpowered study design which did not allow us to perform complex statistical procedures such as multiple imputation. We have used T4 to monitor the safety of the participants only. Finally, in the original protocol, we described that we would add two additional modules on self-harm to the original intervention. In the end, we decided to keep the focus on suicidal thoughts only [39].

### Outcome measures

As part of the feasibility, the system usability scale was used and interviews were conducted. The feasibility was measured at post-test (6 weeks after baseline: T3). *Usability of the online intervention* was measured with the System Usability Scale (SUS). The SUS is composed of 10 statements that are scored on a 5-point scale of extent of agreement (score 0 to 100). The reliability is good (*α* = 0.91) [48]. In our study, the scale also showed good internal consistency (*α* = 0.81). Interventions with scores of 70 and above are accepted as highly usable [48], and scores between 50 and 70 indicate acceptable usability of an intervention. Interventions with scores 50 and below are subject to concerns about their usability by the target population and should be investigated further [48].

In order to answer the research question about the indications of effectiveness, online questionnaires were used. The primary outcome measure in this study is the reduction in the frequency and intensity of *suicidal thoughts*. This was measured with the BSS [49] at baseline (T0), at 2 and 4 weeks into the intervention (T1 and T2) and at T3. The BSS is a 21-item measure assessing the severity of the suicidal ideation [49–51]. Each item is scored from 0 to 2. The total score is obtained by adding the first 19 items and ranges from 0 to 38. High score represents high suicidal ideation. The BSS has good psychometric properties in English [49–51] and in Turkish [52, 53]. In our study, the scale had excellent internal consistency (*α* = 0.92).

Secondary outcome measures are measured at T0 and at T3 and included the following: depression, hopelessness, worrying, quality of life, self-harm behaviour and suicide attempt, and acculturation.

*Depression* was measured with the Beck Depression Inventory (BDI) consisted of 21 items [54]. Each item is scored from 0 to 3. The severity ranges from minimal depressed (score lower than 13) to severely depressed (scores between 29 and 63) [54]. It is a reliable and valid [54] measure for assessing depression. The BDI has been validated in Turkish and Dutch populations [55]. In our study, the scale had good internal consistency (*α* = 0.82).

*Hopelessness* was measured with the Beck Hopelessness Scale (BHS) and contains 20 true and false statements [56]. Each statement is scored from 0 to 1 and the total score ranges from 0 to 20. A high score indicates a high degree of hopelessness. The instrument has good psychometric properties [57, 58]. In our study, the scale showed excellent internal consistency (*α* = 0.92).

*Worrying*: The Penn State Worry Questionnaire (PSWQ-PW) is a 15-item inventory assessing the weekly status of pathological worry [59]. Each item is scored on a 7-point rating scale, ranging from never 0 (never) to 6 (almost always). The total score ranges from 0 to 90 with a high score indicating more worrying. PSWQ-PW shows good reliability and convergent validity [59]. The Turkish version demonstrated good reliability [60]. Additionally, in our study, the internal reliability was good (*α* = 0.78).

*Quality of life*: The Euro Quol (EQ-5D) is an instrument measuring health quality of life and has 5 items: mobility, self-care, usual activities, pain/discomfort and anxiety/depression [61]. Each item is required to be rated as 1 (no problem), 2 (some problem) or 3 (extreme problem). The current health state is also rated on a scale ranging from 0 (worst imaginable state) to 100 (best imaginable state). Both Dutch and Turkish versions have been validated [62, 63]. In our study, the scale showed sufficient internal consistency (*α* = 0.60).

*Suicide attempt and self-harm* (SASH): Four questions measuring the previous suicide attempt and the presence of self-harm were taken from the original Self-Harm Questionnaire [64]. The original scale showed good psychometric properties [64, 65]. In our study, the scale items showed sufficient internal consistency (*α* = 0.60).

*Acculturation* was measured with an adapted version of the Lowlands Acculturation Scale (LAS) representing the difficulties that migrants might face [66] On a 6-point rating scale, item scores range from 1 (not applicable) to 6 (very applicable). The instrument is validated among Turkish migrants living in the Netherlands [66]. It has been adapted to measure the 2 dimensions of acculturation: participation (4 items) and maintenance (11 items) [67, 68]. The subscale participation measures tendency to participate in social life of the host country such as interacting with other minority and majority groups (score range 4 to 23). The subscale maintenance measures the tendency to maintain one’s culture of origin such as preferring to interact with people from the same ethnic background (score range 11 to 60). Higher scores indicate a greater degree of participation and maintenance. The new subscales were reliable and internally consistent in Turkish migrant populations [68, 69]. In our study, both subscales showed good internal consistency (*α* = 0.82 for both subscales).

### Analyses

We defined *feasibility* as engagement with the intervention [33] and its *usability* in daily life, as well as the potential for delivering a full trial in the future. The following components were assessed during the interviews in order to identify the facilitators and barriers influencing the engagement with the intervention: *cultural relevance* (i.e. familiarity and relevance of the therapeutic content to one’s cultural background), *cultural appropriateness* (i.e. appropriateness of the therapeutic content in terms of the cultural context) and *acceptance* (i.e. feedback on the experience of using the intervention in real life) [26].

Telephone interviews were conducted with the participants who completed the intervention and consented to be interviewed (*N* = 12) and started with an open question: ‘What was your overall experience during your participation?’ A topic guide was used for the remainder of the interview which was created on the basis of the relevant literature and discussions with the rest of the researchers taking part in the study (see Additional file 1: Appendix A for the topic guide). The interviews were approximately 30 min long and were recorded for verbatim transcription.

We used thematic analysis as a means of identifying, analysing and reporting explanatory models, and for understanding which elements of the intervention facilitated or hindered participants’ progress during their participation and how the intervention can be optimised to increase its acceptability, relevance and user-friendliness [70]. Pseudonyms were assigned to each interviewee. First, the code system (and categories and themes developed on the basis of the coding process) was developed gradually and collaboratively. The code system was developed on theoretical grounds and included the following categories: definition of facilitators and barriers, specific examples for cultural relevance, appropriateness and acceptability of the intervention and specific recommendations for further improvement. Each of these categories had a number of sub-categories and codes. The definition of therapeutic gains as a result of using the intervention (e.g. therapeutic alliance) emerged during the analysis. This category was considered as important and decided to be analysed separately. The coding system was developed by the first author and was checked independently by a second person (Y.A.) who was not involved in the research. Once an agreement was reached, they were further developed, refined and applied to the transcripts. The first author was the main coder and Y.A. involved as a second coder, who systematically counter-checked the coding, to assure the robustness and the internal validity. The data was coded manually. Disagreements over the coding were discussed between the main and the second coder and where necessary experts (S.R.) were consulted. Detailed descriptive accounts were produced for each major theme alongside the related extracts from participants’ transcripts. Analysis continued until no new themes emerged from the transcripts (see Table 4 for the themes).

The RCT was carried out in accordance with the CONSORT guidelines (see CONSORT checklist). First, *t* tests and chi-square tests, as implemented in SPSS, were used to compare the baseline characteristics of those who were allocated in the intervention group with those who were allocated to the waitlist control group. Second, we tested for the likely effects of the intervention compared to the waitlist control group. We used Bayesian Repeated Measured ANOVA as implemented in the JASP (version 0.9.2), which is a free and open-source graphical programme for statistical analysis. The rationale for choosing the Bayesian approach over the classical inferential approach is based on the following advantages: Bayesian approach considers all possible models (null model, between and within group differences) and assigns more weight to those models that predict data relatively well [71, 72]. The classical inferential model selects the best model, estimates its parameters and might produce overconfident conclusions on data by neglecting model uncertainty [71, 72]. Since the present study design is underpowered, the Bayesian approach accounts for the uncertainty of the all possible models and allows us to make more reliable conclusions based on the existing data [71]. Additionally, we did a sensitivity analysis on those participants who reported severe suicidal ideation at baseline to see if the indications for an effect of the intervention were stronger than in the whole group including those with very mild suicidal ideation.

## Results

### Participants

**Fig. 1:**
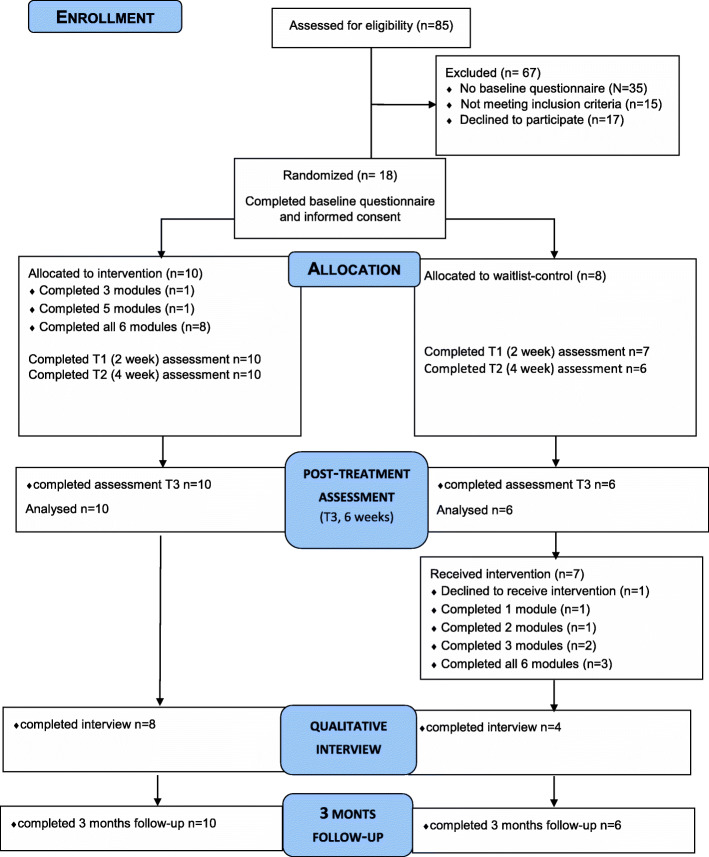
Participant flow through the trial

Figure 1 shows the participant flow through the trial. A total of 85 people registered to the study website, while 50 people completed the baseline questionnaire. Of those, 15 (30%) proved to be ineligible, mainly due to not having suicidal thoughts (*N* = 6, 12%), not living either in the UK or in the Netherlands (*N* = 6, 12%) or being younger than 18 at the time of the registration (*N* = 3, 6%).

The remaining 35 people were eligible but 17 of them (49%) did not return their consent. The remaining 18 (51%) eligible respondents returned their consent forms and were randomised. We contacted all 18 participants for telephone interviews after ending the intervention. Two thirds (*n* = 12; 66.7%) agreed.

Table 1 displays baseline characteristics for all participants randomised. The majority was unemployed (*N* = 11, 61.1%). Out of (*N* = 7, 38%) employed participants, the majority (*N* = 5, 71%) was female. The participants were mostly single (*N* = 11; 61.1%), mainly based in the UK (*N* = 16; 88.8%) and half had a University degree (*N* = 8; 44.4%). The mean age of the total sample was 33.5 years (SD = 8.38). Half of the participants indicated not receiving any form of care at baseline (*N* = 10; 55.5%), while some were seeing a psychologist (*N* = 3, 16.6%), a GP (*N* = 3, 16.6%) or a psychiatrist (*N* = 1, 5.5%). A considerable number of participants were extremely dissatisfied with the previous psychological help (*N* = 11, 61.1%). All but one (5.5%) evaluated the previous help as helpful. There was a significant difference between intervention and waitlist control group participants on the level of acculturation. Participants in the waitlist control group had higher scores on maintenance of their culture of origin compared to the participants in the intervention group (*p* < 0.01).

**Table 1:** Baseline characteristics

Characteristics	Intervention participants (*N* = 10)	Waitlist control participants (*N* = 8)	Total (*N* = 18)	*p* value*
Age (*M*, SD)	34.70 (3.81)	32.00 (9.35)	33.50 (8.38)	0.51
Gender, male (*N*, %)	3 (30%)	2 (25%)	5 (27.7%)	0.81
Type of recruitment (*N*, %)				0.47
Facebook	8 (80%)	5 (62.5%)	13 (72.2%)	
Through a friend	2 (20%)	2 (25%)	4 (22.2%)	
Through a newspaper add	0 (0%)	1 (12.5%)	1 (5.5%)	
Employment status (*N*, %)				0.96
In a paid employment	3 (30%)	2 (25%)	5 (27.7%)	
In an unpaid internship	1 (10%)	1 (12.5%)	2 (11.1%)	
Unemployed	6 (60%)	5 (62.5%)	11 (61.1%)	
Relationship status (*N*, %)				0.65
Single	7 (70%)	4 (50%)	11 (61.1%)	
In a relationship	0 (0%)	1 (12.5%)	1 (5.5%)	
Married	2 (20%)	2 (25%)	4 (22.2%)	
Widow	1 (10%)	1 (12.5%)	2 (11.1%)	
Education level (*N*, %)				0.86
Secondary school	2 (20%)	2 (25%)	4 (22.2%)	
University	5 (50%)	3 (37.5%)	8 (44.4%)	
Others	3 (30%)	3 (37.5%)	6 (33.3%)	
Help-seeking status (*N*, %)				0.39
No help	7 (70%)	3 (37.5%)	10 (55.5%)	
GP	1 (10%)	2 (25%)	3 (16.6%)	
Psychologist	2 (20%)	1 (12.5%)	3 (16.6%)	
Psychiatrist	0 (0%)	1 (12.5%)	1 (5.5%)	
Others	0 (0%)	1 (12.5%)	1 (5.5%)	
Satisfaction with previous help (*N*, %)				0.15
Extremely unhelpful	8 (80%)	3 (37.5%)	11 (61.1%)	
Neither helpful/unhelpful	2 (20%)	4 (50%)	6 (33.3%)	
Helpful	0 (0%)	1 (12.5%)	1 (5.5%)	
Acculturation (*M*, SD)				
Participation	13.00 (4.57)	15.50 (3.42)	14.11 (4.18)	0.21
Maintenance	35.20 (6.26)	43.87 (4.05)	39.05 (6.87)	0.004*
Suicidal ideation (*M*, SD)	12.30 (8.48)	14.87 (8.07)	13.50 (8.07)	0.52
Suicide attempt and self-harm (*M*, SD)	2.10 (1.91)	1.50 (1.69)	1.83 (1.79)	0.50
Depression (*M*, SD)	26.80 (9.73)	33.17 (10.17)	29.61 (10.16)	0.19
Hopelessness (*M*, SD)	12.50 (4.08)	13.50 (4.98)	12.94 (4.39)	0.64
Worry (*M*, SD)	63.50 (19.92)	72.00 (4.05)	67.27 (17.61)	0.32
Quality of life (*M*, SD)	8.30 (1.63)	7.62 (1.40)	8.00 (1.53)	0.36

*p values are based on *t* test or Pearson *X*^2^ test

On average, participants experienced mild levels of suicidal thoughts (*M* = 13.50, SD = 8.07). Out of 18 participants, 7 scored 20 and higher on the BSS, indicating severe suicidal thoughts (38.9%) [50], and 11 (61.1%) scored below 20 on the BSS indicating mild suicidal thoughts. There were substantial levels of depression (*M* = 29.61, SD = 10.16), hopelessness (*M* = 12.94, SD = 4.39) and worry symptoms (*M* = 67.27, SD = 17.61). There were no statistically significant differences at baseline between the two conditions regarding these clinical characteristics.

### Safety

We monitored all the participants carefully through guidance and assessments (t1 and t2: bi-weekly assessments; t3: post-test; t4: 3-month follow-up). We did not call any of the participants as none of the participants exceeded the cut-off score of 29 on the BSS at any time during their participation. Thus, the safety protocol was never activated. There were no suicide attempts or suicides during their participation in the study (Table 2).

**Table 2:** Mean changes from baseline to post-test and follow-up (*N* = 16)

	Intervention (*n* = 10)		Control (*n* = 6)	
	Pre-test	Post-test (6 weeks)	Pre-test	Post-test (6 weeks)
BSS (suicidal ideation; *M*, SD)	12.30 (8.48)	6.10 (4.50)	12.33 (7.60)	6.83 (3.86)
BDI (depression; *M*, SD)	26.80 (9.73)	16.10 (6.96)	32.50 (10.84)	25.10 (8.67)
PSWQ (worrying; *M*, SD)	63.50 (19.92)	58.40 (17.24)	73.33 (15.34)	58.17 (15.52)
SASH (suicide attempt and self-harm) (*M*, SD)	2.10 (1.91)	0.90 (1.59)	0.83 (1.32)	0.83 (1.16)
BHI (hopelessness; *M*, SD)	12.50 (4.08)	7.80 (3.67)	12.66 (5.46)	10.83 (4.26)
EQ5SD (quality of life; *M*, SD)	8.30 (1.63)	7.90 (2.28)	7.33 (1.21)	6.83 (1.16)

### Feasibility

#### Quantitative findings

##### Acceptability

Overall, 8 out of 10 intervention participants (80%) and 3 out of 8 participants (37.5%) in the waitlist control group completed all the sessions (see Fig. 1). Out of 6 sessions, the average number of the completed sessions in the intervention group was *M* = 5.6 (SD = 0.9), while this was *M* = 3.4 (SD = 2.3) for the waitlist control group.

##### Usability

Participants in the intervention group reported an average score of *M* = 36.20 (SD = 5.84) on the System Usability Scale. Participants in the waitlist control group scored *M* = 29.16 on average (SD = 6.52). Both scores are below the cut-off of what might be considered a useful intervention.

#### Qualitative findings

The thematic analysis on the 12 interviews identified 3 overarching themes (see Table 3).

**Table 3:** Themes related to views on the internet intervention (Kıyma Canına) and its adapted content *N* = 12 (9 women, 3 men) of Turkish descent, and aged 23–56, in the Netherlands and in the UK

Themes	*N* = 12, *N* (%)
1.Therapeutic change	12 (100)
Therapeutic alliance	9 (75)
Self-management	10 (83)
2.The gap between reading it and doing it in real life	7 (58)
Feeling connected	7 (58)
Not feeling connected	5 (42)
3.Recommendations for improvement	12 (100)
More diversity	9 (75)
More directive approach	8 (67)

##### Theme 1: Therapeutic change

Therapeutic alliance

All participants commented on developing therapeutic alliance (i.e. the relationship between coaches and the participants) as a result of using the intervention. Many participants identified *personalised feedback* as one of the key components helping them to benefit from the intervention. For some participants, personalised feedback did not only motivate them to continue but it also provided a safe environment to disclose their experiences:

Receiving feedback was like exchanging letters with someone….Sometimes you cannot talk to everyone about certain things. But receiving feedback and being able to respond to it, was like a relief …. As I went through them, I kept on discovering new things about myself Participant A.

Self-management

Several participants were going through important life events, for instance, domestic violence, loss of a loved one and work-related stress which resulted in crises. Crisis was often defined as *feeling confused* about how to handle stress. The psycho-educational aspect of the exercises and the feedback helped them to understand these crisis situations:

I was going through a trauma….and was not able to make much sense of what was happening to me…the feedback helped me to make sense of it all. It helped me to explain things from a scientific point of view Participant D

Almost all participants emphasised *better self-management* as one of the most important benefits of the intervention. Several participants mentioned that the exercises about worry time were helpful in terms of managing the crisis situations. More specifically, worry time helped them to feel more in control of their thoughts and this was felt as an important *accomplishment*. Many participants emphasised this feeling as an important source of motivation:

Sometimes when I was panicking about something, I was letting myself to worry…to think of the worst case scenario that could happen to me… I was thinking of that for 10 minutes or so and afterwards I was able to feel better…..Managing to do that was really helpful….. Participant F

It [following the intervention] gave me some peace of mind as I was doing something at least Participant H

##### Theme 2: The gap between ‘reading it’ and ‘doing it’ in real life

Feeling connected

Feeling connected with the intervention and/or with the personal coach appeared as a strong facilitator. Being able to relate to the content helped them feel connected with the intervention. Suitability of the intervention was emphasised as a strong facilitator for feeling connected:

I felt the exercises were suitable with my life style…Working with a coach was also helpful in terms of feeling connected… I feel I gained skills that I could use for the rest of my life Participant A

Some participants named mindfulness and mentalisation exercises as the most helpful ones in terms of their recovery. The most commonly mentioned reasoning was that such exercises were not restricted with the *context* and were easy to follow during the day:

Mindfulness exercises [imagining your thoughts as if they were clouds and watching them pass by] are not restricted with the context…so you can do them when you are sitting at the office or when you are doing yoga….. Participant D

All participants spoke about feeling familiar with the culturally adapted content such as cultural case examples and the well-known metaphors explaining psychological distress and crisis situations. Several participants identified *cultural familiarity* as a pleasant experience helping them to feel more connected with the intervention. Those who feel connected were also able to relate to the intervention (i.e. *cultural relevance*) and often found it appropriate (i.e*. culturally appropriate*):

The intervention was very familiar and it felt like I was not only getting professional help but was also talking to a friend Participant E

I think all the examples were appropriate to the Turkish culture…They were also representative of the types of problems that migrant populations are likely to face Participant G

In terms of the acceptability of the intervention, participants expressed contradictory opinions. For some of them, the ‘self-help’ principles made it difficult to use the intervention. They emphasised that guidance and cultural adaptations were not sufficient:

The difficulty with the online therapy is that, we need to do things on our own. When you see a psychologist….when there is a person in front of you….you feel more in control….I think there should be a psychologist in front of you and you should feel pushed….Do you see my point? After all not feeling in control is the main reason why we need psychological help….Isn’t it? Participant H

Not feeling connected

Those who did not consider themselves as having severe suicidal ideation did not feel connected with the intervention. They often reported feeling uncomfortable when they thought they were being considered as a severe case:

The intervention was for severe cases [people who have intense thoughts about suicide]….I am not in that group….. so sometimes the questions and the exercises were not so relevant to me. I asked myself if this is how they really think about me. Am I considered as a “nut case”? This was affecting my willingness to participate….You know…how you feel changes your decisions…. Participant H

Not feeling connected was also mentioned in relation to the culturally adapted content. Some participants mentioned that the adaptations, such as cultural case examples, were representative of a group of traditional people and they were not able to relate to this specific group. They were more ambivalent especially about the appropriateness of the cultural case examples (i.e. adaptations in the context of the intervention):

I don’t know…..Someone’s daughter broke up with her fiancé and so and so forth [cultural case example]…I only laugh at such things when I hear them…they do not fit with my philosophy… Participant B

The cultural case examples attracted my attention….Because my life experiences are different, I felt awkward sometimes Participant C

##### Theme 3: Recommendations for improvement

More diversity

Many participants recommended to include more diversity in the context (e.g. more case examples representing different backgrounds). All participants commented on the usefulness of the online diary, which was part of the crisis plan encouraging participants to monitor their suicidal thoughts on a daily basis. For many participants, online diary was frustrating as it was not representative of the variety of feelings they experience throughout the day and was also not practical:

I struggled with the online diary…. I was asked to upload a picture representing my typical mood of the day….But finding a specific picture representing a specific feeling was not really feasible for me…..The options for feelings were also too generic. I feel a variety of emotions during the day not only sad, angry or happy…..I felt I was not able to express myself there Participant C

More directive approach

Several participants spoke about including more instructions and coaching in the sessions. This was often mentioned as a way of feeling more in control while implementing the intervention in their daily life:

There were many exercises…..and I needed to find out which one works better for me…. I didn’t quite catch that in the beginning…It worried me… I felt I was not in control….I think there could be more personalised guidance so that it’s easier to find the right exercises Participant I

### Indications for the likely effects

**Fig. 2:**
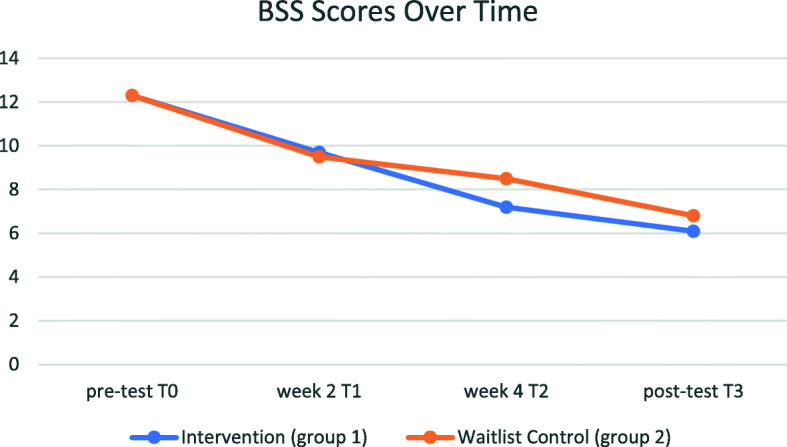
The interaction plot between group status and time (pre-test, week 2, week 4 and post-test) indicating changes in suicidal thinking in the intervention group when compared with the waitlist control group

Overall, the analyses showed indication for change in suicidal ideation, hopelessness and depression scores over time, but not for worrying and quality of life scores (see Table 2). These improvements occurred in the intervention group as well as in the control group and there were no post-test differences between the two groups (Fig. 2).

A Bayesian two-way repeated measures ANOVA comparing the pre- (T0) and post-test (T3) BSS scores of intervention and waitlist control group participants revealed indication for a time effect on suicidal ideation (BF_10_, 50.4), hopelessness (BF_10_, 14.44) and depression (BF_10_, 127.09) scores, but not for group or interaction effects (see Table 4).

**Table 4:** Model comparisons between the null and the alternative models for the study variables (*N* = 16)

Variable	Model	BF10	Ratio
BSS (T0 vs T3)	Null model	1.00	a
	Time	50.41	1.00
	Group	0.46	0.01
	Time + group	27.27	0.54
	Time + group + time × group	11.89	0.23
BSS (T1 vs T2)	Null model	1.00	a
	Time	1.32	1.00^b^
	Group	0.60	0.45
	Time + group	0.81	0.61
	Time + group + time × group	0.46	0.35
BDI (T0 vs T3)	Null model	1.00	a
	Time	99.84	1.00^b^
	Group	1.06	0.01
	Time + group	127.09	1.27^c^
	Time + group + time × group	70.34	0.70
BHI (T0 vs T3)	Null model	1.00	a
	Time	14.44	1.00^b^
	Group	0.54	0.04
	Time + group	8.30	0.57
	Time + group + time × group	7.27	0.50
PSWQ (T0 vs T3)	Null model	1.00	a
	Time	2.85	1.00^b^
	Group	0.57	0.20
	Time + group	1.72	0.60
	Time + group + time × group	1.29	0.45
EQ5SD (T0 vs T3)	Null model	1.00	a
	Time	0.43	1.00^b^
	Group	0.77	1.79^c^
	Time + group	0.33	0.76
	Time + group + time × group	0.14	0.32

T0, baseline (pre-test); T1, bi-weekly measures of BSS at week 2; T2, bi-weekly measures of BSS at week 4; T3, post-test; BF10, Bayesian factor grading the intensity of the evidence supporting the alternative model against the null model. Ratio: This column represents the ratio (the likelihood) of the effect of time against the group, time and group and the interaction models. The time model is the denominator. The BF10 of each model has been divided by the BF10 of the time model in order to calculate the ratio of each model when compared with the time model

^a^The ratio for the null model was irrelevant

^b^The ratio of time against time is always 1

^c^When the ratio is between 0 and 1, there is a weak evidence supporting the alternative model against the time model. When it is greater than 1, that means there is a stronger evidence supporting the alternative model against the time model

The interaction plot for the suicidal ideation scores (see Fig. 2) indicates slightly more improvement in suicidal ideation among those in the intervention group compared to those in the waitlist control group. However, this was not supported by the results of the Bayesian analyses.

We have repeated the Bayesian two-way repeated measures ANOVA on those who scored 20 and above on BSS scores only (*N* = 7). The results indicated stronger indication for a time effect on suicidal ideation, and depression scores, but not for group or interaction effects (see Table 5 in Additional file 1: appendix C). There were two exceptions. Among those with severe suicidal ideation, there was stronger indication for time and group effects on hopelessness scores (BF_10_, 10.512). This means that it is strongly likely that among those with severe suicidal ideation in both groups, the hopelessness scores were improved over time. There was also strong indication for an interaction effect on the quality of life (BF_10_, 8.176). Among those with severe suicidal ideation in the intervention group, there is strong indication for a greater improvement in the quality of life compared to those with severe suicidal ideation scores in the waitlist control group.

## Discussion

In the current study, we included 18 participants and among those, 7 of them had substantial levels of suicidal thoughts at baseline. Despite this, no one reported a risk for a suicide attempt and the safety protocol did not need to be activated. Although therapeutic benefits of the intervention were emphasised (e.g. feeling connected with the intervention and/or personal coach), the low scores on the usability of the intervention showed a number of barriers compromising its usability in daily life. Those with mild suicidal ideation and those who were not relating to the culturally adapted content (e.g. specific cultural case examples) emphasised not being represented by the content of the intervention. Further, there was no indication that the intervention group leads to better health outcomes than the control group. In both groups, there was a reduction in suicidal ideation, depression and hopelessness, but not in worrying and quality of life.

### Participants in the study

The reasons for the low uptake of the intervention remain unclear. One possible explanation is the difficulty in engaging with the target population during the recruitment process. In line with the trial of Ünlü İnce and colleagues [73], various channels have been used during the recruitment process such as social and mainstream media promotion and TV programmes. Additionally, public events have been organised such as exhibition and film discussion. The rationale for using the latter methods was the growing evidence supporting the value of them in engaging with the target group concerning topics related with stigma and shame [74]. Even though such events were organised in collaboration with community organisations and were usually well-attended, they did increase the publicity but did not often result in more participants.

Lack of anonymity during the recruitment process might be another important barrier. As part of the safety protocol, we collected personal information (name, address) and GP details during the recruitment process. Qualitative evidence on cultural meaning of suicide among Turkish migrants indicates that disclosing suicidal thoughts might mean dishonouring family and community by failing their expectations [11, 23]. The majority of the potential participants were lost during the recruitment process mainly because they did not want to fill in the baseline questionnaire and/or send their personal information (*N* = 35). Thus, it could be that the fear of disclosing identity might not have been eliminated during the recruitment process. The paper consent procedures in this current study might have contributed to this fear.

Further, it might also be that our target population was reserved in terms of seeking help for suicidal thoughts. Although the risk for suicidal behaviours is well-documented among Turkish populations [12], their mental health literacy (i.e. recognising suicidal thoughts and symptoms of psychological distress) might be low. Support for the deleterious impact of the low mental health literacy on participant engagement comes from a recent pilot study investigating the feasibility of a mobile app in treating depression among Hispanic population in the States [37]. Low mental health literacy was identified as a barrier restricting the uptake of the online app in this community (37). Although appealing, the mental health literacy was not investigated in our study. Thus, its impact in the implementation of our intervention remains unclear.

There might be other human factors contributing to the low uptake of the intervention. For instance, the target population might not have wanted an online intervention. The negative general perception of online interventions (e.g. they might be more appropriate to people from higher social class or those who are younger or more educated) was an important barrier hindering the engagement with the target population in a pilot RCT in Lebanon [75]. Another attitudinal barrier might be about participating in an experimental study (with lots of extra questionnaires and the chance of entering the control group). Participating in research might precipitate the fear that their personal information might be used against them by the institutions of the host countries such as not hiring them for a job position because of having suicidal thoughts or symptoms of depression [73].

### Feasibility

The low usability scores pointed to the barriers during the implementation of the intervention in real life. During the semi-structured interviews, not feeling represented by the content of the intervention was identified by some participants despite the cultural adaptation of the intervention content. Tseng has argued for ensuring philosophical adaptations, that is the meaning of therapy and a therapeutic relationship, alongside technical, theoretical and practical modifications [76]. More specifically, the characters representing Turkish migrants in the case examples were defined as more traditional in adherence to cultural and religious practices than the participants, and less educated than some participants. It could be that some of the participants in our sample had different cultural values and norms, emphasising caution in cultural adaptations to suit a range of demographic influences and cultural transition or acculturation.

The main reason for contextualising the original intervention according to the Turkish cultural norms and values was to reach out to those who are more attached to these values (i.e. more traditional) in particular. Research from the Netherlands [5, 6, 44], Belgium [77] and Germany [7] emphasise that Turkish migrants who are more traditional are more at risk for suicidal behaviour and are less likely to access the available treatment [77, 78]. It could be that the online form of delivery of the intervention was not suitable for this group specifically or that the groups in the Netherlands are more traditional than the majority of participants who were from the UK. This necessitates a measure of acculturation in future research studies and adaptation to suit a variety of cultural groups.

There is a preliminary evidence for the acceptability of a culturally adapted group intervention targeting first-generation Turkish migrants in the UK [79]. Similar to the content of our online intervention, Perry and colleagues adapted the context of their intervention by incorporating idioms of distress and cultural case examples [79]. Their intervention was delivered in a group format in a community setting which was accessible especially for the first-generation migrants [79]. Since the majority of participants considered the adaptations appropriate to the therapeutic needs of the traditional group of participants, it could be that not the content of adaptations but the format of delivery restricted the engagement with this group of participants.

It could also be that in our study, the cultural adaptations on the context of the intervention underscored the diversity of the Turkish migrant populations in Europe. This finding points to the ‘overgeneralisation’ as one of the main challenges encountered in the cultural adaptation literature [26]. Theoretically, it could be argued that even though migrants might share the same cultural identity (e.g. ethnicity, nationality and social group), there might be differences in micro-identities (e.g. political views, religion, sexual orientation) embedded within their cultural identities [80]. The settlement and acculturation processes might complicate the formation of these identities even more so when the transnational migrant populations, such as Turkish migrants, are concerned. For instance, there has been an increase in political refugees from Turkey in West Europe (e.g. Germany, the Netherlands, and the UK) in recent years [81]. This new group of Turkish migrants might have different micro-identities compared to the Turkish migrant populations with a long-standing history of settlement who have strong ties with their heritage culture [82]. Even though we do not have information about the migration history of the participants, it might be that our sample was more representative of the recent group and this might have restricted the usability of the intervention in their daily life. Thus, a sub-group needs to be better identified for maximal benefit of e-mental health interventions. Importantly, those with more traditional background may prefer alternatives such as face-to-face and/or combination of e-mental health and face-to-face delivery (blended care); therefore, e-mental health interventions alone may not address the needs of all.

Another reason for feeling not represented was identified as not having severe suicidal thoughts. It could be that our intervention was more suitable to those with more severe suicidal ideation. Support for this comes from the results of our sensitivity analyses. We found stronger indications for an improvement in suicidal ideation, depression and hopelessness scores in both groups when we restricted the analyses with those with severe suicidal ideation scores. We also found stronger indication for a greater improvement in quality of life among those with severe suicidal ideation in the intervention group compared to those with severe suicidal ideation in the waitlist control group. These findings suggest that our inclusion criteria (scoring 1 and above on BSS) were too broad. This is discrepant from the trial of van Spijker and colleagues [30] where severe levels of suicidality and depression were associated with poor motivation [30]. These differences could be explained with the cultural differences in help-seeking patterns. For instance, it is well-known that Turkish migrants tend to delay help-seeking until the symptoms of distress are more severe [67, 83]. It could be that when the psychological distress caused by suicidal thoughts is unbearable then stigma and shame attached to suicide might have less influence on their motivation to seek help. However, this remains inconclusive for a number of reasons. First of all, we did not measure the mental illness stigma among participants. We also did not investigate whether there was a relationship between the intensity of suicidal ideation, stigma and help-seeking for suicide among participants. Additionally, we did not have a sufficient number of participants to consider this trial definitive and assess effectiveness.

Feasibility issues might also be related with the delivery of the intervention. Some participants recommended a more directive approach giving them more clear instructions in following the sessions. Our intervention was set out with a lot of different exercises and participants were encouraged to choose the appropriate ones for themselves. It might be argued that more choice is not always the best option especially for some ethnic groups who are seeking explicit advice and assertion in their help-seeking process [84].

### Indications for the likely effects

One of the obvious reasons for not finding an indication for the effect of the intervention was the small sample size. However, since the scores of the two groups were almost identical, we do not expect that a larger trial would have revealed effects. Both groups improved over time.

The finding that there was an indication for an improvement in suicidal ideation scores and in depression in the control group is in line with the results of the other trials [30, 32]. One of the explanations could be the effect of the safety protocol itself (i.e. being measured bi-weekly, having a safety procedure installed when necessary) in the waitlist control group during the first 6-week period upon randomisation. It could be that safety protocol functions as an intervention in itself in the waitlist control group and might need to be separately investigated. There is growing evidence indicating the value of safety planning as a standalone psycho-educational intervention increasing awareness about crisis situations, warning signs and available services for further help [85].

Alternatively, improvements in both groups can be attributed to receiving usual care which cannot be ruled out in the present study. Even though the majority of participants in both groups indicated not receiving any other help at baseline, their exposure to other sources of information and/or help was not carefully monitored throughout the study. This is an important methodological limitation [30, 32]. Future studies could benefit from an attention-control condition to monitor the elements and effects of usual care [30]. Another possibility might just be the passing of time in that many states of distress is self-limiting and people might just spontaneously recover [86].

Notwithstanding with these limitations, this study has various strengths. An important strength is the safety of our intervention. The safety protocol did not need to be activated because none of the participants exceeded the cut-off score 29 on the BSS. None of the participants reported any adverse effects of the intervention, such as increased intensity of suicidal thinking, as a result of their participation. The safety of the e-mental health intervention is in line with the other trials investigating its effectiveness in general populations in the Netherlands [30], in Belgium [32] and in Australia [31]. Thus, vulnerable groups such as minority groups with severe mental health symptoms could be studied safely.

Another strength is that the study followed a particular theoretical framework and a systematic approach to adapt the intervention according to the cultural beliefs and attributions about suicide and help-seeking for suicide among this high-risk group. The challenges encountered during the adaptation process, such as overgeneralisation, highlight the importance of investigating the components of effective cultural adaptation further. For instance, when the context of the intervention is adapted for a particular sub-group within the target population, the fidelity of the intervention might be compromised; therefore, it might not be effective.

It is also noteworthy that the majority of the participants emphasised the therapeutic effect of guidance. Guidance was often considered as helpful in terms of feeling connected with the intervention. One of the notable recommendations of the participants was about incorporating more guidance in the intervention. The expectation for a more directive approach is in line with the cultural adaptation practices for Muslim populations [84]. The refusal to give explicit advice or lacking assertion has been associated with incompetence and indecisiveness of the mental health professional, which might lead to the patient becoming irritated or discontinuing their therapy [84]. This finding supports the rationale of the decision for incorporating guidance into the e-mental health intervention in the present study as being different to the other trials [30, 32, 87].

## Implications and conclusions

Overall, the present study is an important step to further the current knowledge on whether online interventions could provide a feasible and an effective alternative in more complex contexts, including ethnic minority groups who are at elevated risk for suicidal behaviours. To improve engagement with Turkish migrant populations in their help-seeking process, the corresponding author is in progress of developing and piloting a brief community-based anti-stigma intervention in partnership between Derman, a non-governmental organisation providing bi-lingual psychological and advocacy services to Turkish-speaking migrants, and the East London NHS Foundation Trust in the UK. If feasible and acceptable, it is planned to offer it as a first-line intervention to improve suicide literacy. The second step will be to offer the refined online intervention to those with severe levels of suicidal thoughts and depression symptoms. This design is also in line with the Medical Research Council Framework (MRC) to test complex psychological interventions [88]. Additionally, including another arm offering a face-to-face treatment and/or a blended care within the design of future studies could make the participation more appealing to those who have strong preferences for face-to-face treatment over e-mental health.

In terms of the content of the intervention, cultural adaptations should not exclusively represent the norms and values of the settled community but also the values of those who are in the process of settlement. Another key lesson for optimising the intervention further for the Turkish migrant populations is to structure the content of the intervention more. This might be important especially for ethnic groups who seek for instructions in their help-seeking process [89]. Since the intervention was safe to use in our study, as well as in other trials, the intervention can be embedded within anonymous online platforms such as Samaritans in the UK and 113 online in the Netherlands. This might remove barriers, for instance, fear of disclosing identity and fear of dishonouring one’s family, which were highlighted as potential barriers hindering participation in the present study [89]. Since the participants valued the process of guidance, the online intervention might be well suited as an add-on intervention to regular psychotherapeutic treatment. The effectiveness of such an additional intervention to regular face-to-face treatment should be studied carefully.

To conclude, the challenges encountered in this feasibility study can be viewed as part of the incremental steps necessary to build future success in implementing online interventions among ethnic minorities in treatment of suicidal behaviour. This study and the previous trials seem to suggest the intervention needs further work and refinement and there should be more feasibility and exploratory trials of a modified intervention, refine it iteratively with feedback.

## Supplementary Information

**Additional file 1.** Appendices A, B, C, and D

**Additional file 2.** CONSORT checklist

## Data Availability

The datasets generated and/or analysed during the current study are not publicly available due to them containing information that could compromise research participant privacy/consent but are available from the corresponding author on reasonable request with permission of the Department of Clinical Psychology, VU Amsterdam University.

## Abbreviations

CBT: Cognitive Behavioural Therapy
DBT: Dialectical Behavioural Therapy
RCT: Randomised controlled trial
PC: Personal computer
GP: General practitioner
QMERC: Queen Mary Medical Ethical Research Committee
BSS: Beck Suicidal ideation Scale
BDI: Beck Depression Scale
BHS: Beck Hopelessness Scale
PSQ-PW: The Penn State Worry Questionnaire
EQ-5D: The Euro Quol
SASH: Suicide attempt and self-harm
LAS: Lowlands Acculturation Scale
