# Supplementary material for: Reducing suicidal ideation among Turkish migrants in the Netherlands and in the UK: the feasibility of a randomised controlled trial of a guided online intervention
Source: Pilot Feasibility Stud. 2021 Jan 25;7:30. doi: 10.1186/s40814-021-00772-9 (PMC7830826; doi:10.1186/s40814-021-00772-9)
Supplement: Supplementary file 1 — Additional file 1. Appendices A, B, C, and D [file 40814_2021_772_MOESM1_ESM.docx]

Appendix A

Qualitative Questions:

1. What was your overall experience during your participation? (What did you like most/ What did you like least? and some specific questions about the features of the platform)
   1. Did you use the online diary? How was your experience?
   2. How was it for you to evaluate your emotions on a scale of smileys (instead of numbers)?
   3. Any other feature of the platform you can name as helpful/unhelpful for you? For instance we have shortened the text and included more visual features in the content of the sessions. How was your experience with these adjustments? Were they useful in following the sessions?
2. Did you feel awkward/uncomfortable whilst using the platform? For instance, we have used cultural case examples to make the content of the intervention more relevant to our participants. How was your experience with these examples?
3. How did you find the language of the course? Was it complicated/ easy to follow/ formal/ informal?
4. Did you find the pictures (in the platform) appropriate and relevant to the course
5. How was your experience with the exercises in the sessions? (What did you like most/ What did you like least?) Could you give an example from your experience?
6. How was your experience with the guidance? Was receiving feedback from your coach helpful? Can you name the helpful and/or unhelpful aspects of the guidance?
7. If you were to make any changes in this course, what would you change? (suggestions for improvement

Appendix B

Summary of the Decisions for Cultural Adaptation

| **Intervention** | **Cultural Adaptation Components** | | | | | |
| --- | --- | --- | --- | --- | --- | --- |
|  | **Linguistic**  **Adaptations** | **Metaphors** | **Concepts** | **Context** | **Goals** | **Methods** |
| **Introduction** | Information: The modules are available in 3 languages (Dutch, English, Turkish) and the relevance and understandability of the content has been checked by native speakers. | Use of the metaphors: “KiymaCanina-Don`t crush your life energy’’ instead of ‘’suicide’’ to name the intervention;  *“don`t give me fish, teach me how I can catch fish”* to participate actively by doing the exercises and homework. | Use of the concepts “*interdependence*” and “*independence*’’ e.g. it is their responsibility to follow the modules (independence) and guidance is available if further assistance is needed (interdependence). | Information: the context of the intervention and exercises are tailored in the light of the previous research with Turkish communities (Eylem et al., 2016) | Information: the goals of the modules are in line with the cultural values. | Information: the methods of the intervention is tailored according to Turkish cultural values. |
|  | **Linguistic adaptations** | **Metaphors** | **Concepts** | **Context** | **Goals** | **Methods** |
| **Module 1**  **Thinking about Suicide** | Reducing literacy, rephrasing the sentences. | None | Use of *religiosity* and *interdependence* e.g. “It is normal to think of death in Islam but thinking of suicide is different” (religiosity), feeling afraid of letting family down (interdependence) | Use of Turkish names (e.g. Tarik and Cemil) and context in case studies of suicidal thinking. | None | Use of modelling (i.e. using case examples) to explain how users can do the exercises;  Some mentalization techniques have been added to the “worry time’’ exercise. |
| **Module 2**  **Dealing with Feelings and Crises** | **Linguistic adaptations** | **Metaphors** | **Concepts** | **Context** | **Goals** | **Methods** |
|  | Reducing literacy, rephrasing the sentences | Use of metaphors to explain the crisis “wanting to bang your head from wall to wall’’, “feeling like your head is going to explode” | Use of the concept “namus” (i.e. honour) in the case study e.g. Fatma is experiencing crisis after she has an argument with her parents about not being allowed to stay over in her friends` place. | Use of Turkish names and context in case studies. | Respecting users` possible concern about sharing their feelings with someone by encouraging them to imagine themselves sharing their feelings with someone in “an experiment in sharing your feelings” | Use of visualization (e.g. happy picture of you, sad picture of you) instead of feeling thermometer; some mentalization techniques have been added to the “sit still and do nothing’’ exercise and the name has been replaced as “sit still and reflect back’’; the information and exercises about self-harm have been removed. |
| **Module 3**  **Automatic Thoughts** | **Linguistic adaptations** | **Metaphors** | **Concepts** | **Context** | **Goals** | **Methods** |
|  | Reducing literacy, rephrasing sentences explaining theoretical background. | None | Use of cultural expression of sadness (e.g. moral bozuklugu) in exercises | Use of Turkish names and context in the case studies; rephrasing automatic thoughts according to the cultural knowledge of such thoughts in Turkish context e.g. I have failed my family, I am a black spot in my family`s forehead (i.e. I have humiliated my family by bringing bad reputation to them) | None | Adding some mentalization techniques to the “feelings of guilt exercise’’ (e.g. sometimes we confuse our thoughts with facts and feel guilty because of our predictions) |
| **Module 4**  **Thinking Habits** | **Linguistic adaptations** | **Metaphors** | **Concepts** | **Context** | **Goals** | **Methods** |
|  | Reducing literacy, rephrasing thinking patterns according to the cultural knowledge of Turkish populations. | Use of the “black and white filter in ohotography” in order to explain thinking patterns e.g. Thinking patterns are like particular type of filters (such as black & white filter) limiting our perception with only one perspective | Use of concepts-honour, namus in the case examples | Use of Turkish names and context in the case studies; giving examples from Turkish cases to explain the thinking patterns e.g. an example for negative thinking style-“Cemil feels upset by his friend’s behaviour when they come across at the supermarket. He might think Everyone in the community knows about my daughter’s break-up with her fiancé.No-one will ever respect me again. Why do I even stay on this earth? If he reacts like this, Cemil might well end up with some thoughts of suicide.” | None | Perhaps not using the “metaphors and exaggeration exercise” as metaphors and exaggerations are embedded within Turkish culture! |
| **Module 5**  **Thinking Differently** | **Linguistic adaptations** | **Metaphors** | **Concepts** | **Context** | **Goals** | **Methods** |
|  | Reducing literacy, rephrasing sentences explaining theoretical background. | None | Use of cultural concepts-namus, man’s pride (honour) whilst rephrasing automatic thoughts | Use of Turkish names and context whilst rephrasing automatic thoughts and counter thoughts  e.g. automatic thought- ‘*No one will ever respect me again’,* counter thought- ‘*Some people do not respect me again’* | The goal of “Does life have any meaning?” exercise has been changed to “Is my life worth living?” exercise. The former had a very general goal but the latter is more specific and it is in accord with the cultural knowledge | Use of modeling in exercises about questioning the automatic thoughts and finding counter thoughts. |
| **Module 6**  **Dealing with Future Setbacks** | **Linguistic adaptations** | **Metaphors** | **Concepts** | **Context** | **Goals** | **Methods** |
|  | Reducing literacy, rephrasing sentences explaining theoretical background. | None | Use of cultural concepts-man`s pride (honour) whilst changing automatic thoughts to counter thoughts in the case examples. | Use of Turkish names and context whilst re-phrasing the case examples | None | None |

Appendix C

Table 5. Model Comparisons Between the Null and the Alternative Models for the Study Variables among Those with Severe Suicidal Ideation Scores on BSS (N=7)

| Variable | Model | BF10 | Ratio |
| --- | --- | --- | --- |
| BSS (T0 vs T3) | Null model | 1.00 | ^a^ |
|  | Time | 2.567.569 | 1.00 |
|  | Group | 0.60 | 0.23 |
|  | Time + Group | 1.840.740 | 0.33 |
|  | Time+Group+Time*Group | 1.140.252 | 0.44 |
| BSS (T1 vs T2) | Null Model | 1.00 | ^a^ |
|  | Time | 2.688.888 | 1.00^b^ |
|  | Group | 0.60 | 0.37 |
|  | Time+Group | 1.889.473 | 0.71 |
|  | Time+Group+Time*Group | 1.155.267 | 0.43 |
| BDI (T0 vs T3) | Null Model | 1.00 | ^a^ |
|  | Time | 5.289 | 1.00^b^ |
|  | Group | 1.105 | 0.21 |
|  | Time+Group | 9.476 | 1.79^c^ |
|  | Time+Group+Time*Group | 5.161 | 0.97 |
| BHI (T0 vs T3) | Null Model | 1.00 | ^a^ |
|  | Time | 6.943 | 1.00^b^ |
|  | Group | 0.933 | 0.13 |
|  | Time+Group | 10.512 | 1.51^c^ |
|  | Time+Group+Time*Group | 6.765 | 0.97 |
| PSWQ (T0 vs T3) | Null Model | 1.00 | ^a^ |
|  | Time | 0.831 | 1.00^b^ |
|  | Group | 0.755 | 0.91 |
|  | Time+Group | 0.691 | 0.83 |
|  | Time+Group+Time*Group | 0.365 | 0.48 |
| EQ5SD (T0 vs T3) | Null Model | 1.00 | ^a^ |
|  | Time | 3.490 | 1.00^b^ |
|  | Group | 0.785 | 0.24 |
|  | Time+Group | 3.387 | 0.97 |
|  | Time+Group+Time*Group | 8.176 | 2.34^c^ |

*Note* T0: Baseline (pre-test); T1: Bi-weekly measures of BSS at week 2; T2: Bi-weekly measures of BSS at week 4; T3: post-test; BF10: Bayesian factor grading the intensity of the evidence supporting the alternative model against the null model

Ratio: This column represents the ratio (the likelihood) of the effect of time against the group, time and group and the interaction models. The time model is denominator. The BF10 of each model has been divided by the BF10 of the time model in order to calculate the ratio of each model when compared with the time model.

^a^: The ratio for the null model was irrelevant

^b^:The ratio of time against time is always 1

^c^: When the ratio is between 0 and 1, there is a weak evidence supporting the alternative model against the time model. When it is greater than 1, that means there is a stronger evidence supporting the alternative model against the time model.

Appendix D

Interaction Plots of Outcome Measures

Figure. The interaction plot between group status and time (pre and post-test) indicating changes in BDI (depression) scores in intervention group when compared with the wait-list control group

Figure. The interaction plot between group status and time (pre and post-test) indicating changes in BHI (Hopelessness) scores in intervention group when compared with the wait-list control group

Figure. The interaction plot between group status and time (pre and post-test) indicating changes in Suicide Attempt and Self-Harm in intervention group when compared with the wait-list control group

Figure. The interaction plot between group status and time (pre and post-test) indicating changes in PSWQ (Worrying) scores in intervention group when compared with the wait-list control group

Figure. The interaction plot between group status and time (pre and post-test) indicating changes in EQ5SD (Quality of Life) scores in intervention group when compared with the wait-list control group
